# Analytical and Finite Element Modeling of Nanomembranes for Miniaturized, Continuous Hemodialysis

**DOI:** 10.3390/membranes6010006

**Published:** 2015-12-31

**Authors:** Tucker Burgin, Dean Johnson, Henry Chung, Alfred Clark, James McGrath

**Affiliations:** 1 Department of Biomedical Engineering, University of Rochester, 252 Elmwood Ave, Rochester, NY 14627, USA; tburgin@ur.rochester.edu (T.B.); dean.johnson@rochester.edu (D.J.); huchung@seas.rochester.edu (H.C.); 2 Department of Mechanical Engineering, University of Rochester, 252 Elmwood Ave, Rochester, NY 14627, USA; aclark@ur.rochester.edu

**Keywords:** hemodialysis, membranes, dialysis membranes, models, diffusion, continuous dialysis, wearable artificial kidney, nanomembranes

## Abstract

Hemodialysis involves large, periodic treatment doses using large-area membranes. If the permeability of dialysis membranes could be increased, it would reduce the necessary dialyzer size and could enable a wearable device that administers a continuous, low dose treatment of chronic kidney disease. This paper explores the application of ultrathin silicon membranes to this purpose, by way of analytical and finite element models of diffusive and convective transport of plasma solutes during hemodialysis, which we show to be predictive of experimental results. A proof-of-concept miniature nanomembrane dialyzer design is then proposed and analytically predicted to clear uremic toxins at near-ideal levels, as measured by several markers of dialysis adequacy. This work suggests the feasibility of miniature nanomembrane-based dialyzers that achieve therapeutic levels of uremic toxin clearance for patients with kidney failure.

## 1. Introduction

Kidney failure is a life-threatening condition characterized by inadequate renal filtration causing increased plasma concentrations of uremic toxins. Patients with chronic or end-stage kidney failure may be treated with kidney transplantation, but are often forced to rely on hemodialysis instead due to the scarcity of donor kidneys. Indeed, in 2012 the active transplant waiting list population exceeded the donor supply by 2.7 times, and the incidence of renal disease continues to rise into hundreds of thousands of new diagnoses per year [1].

Hemodialysis is an artificial supplement or replacement for kidney function that aims to clear uremic toxins and excess water from a patient’s blood by molecular exchange between uremic blood and a buffered solution of salts, sugar, and other critical analytes, the dialysate. Several different incarnations of the treatment exist, but typically the patient’s blood is perfused into a space separating it from the dialysate by a semi-permeable membrane, and toxins are allowed to diffuse through the membrane into the dialysate down their concentration gradients while transmembrane osmotic pressure promotes ultrafiltration to reduce the patient’s total body fluid [2].

While intermittent hemodialysis has been the standard of care for replacing lost kidney function since the mid 1970’s, the therapy has many limitations [3]. The intermittent nature of most dialysis treatments means that dialysis is typically administered in large doses, in an attempt to compensate for toxin generation during the time that the patient is not receiving treatment. Evidence exists to suggest that the resulting "sawtooth" pattern of toxin concentrations (decreasing rapidly during treatment, recovering between treatments, being decreased again, and so on) exacerbates the morbidities of kidney failure when compared to more consistent techniques, owing to the relatively high concentration of toxins over average just before treatment [4].

Perhaps unsurprisingly, increased patient morbidity and mortality have been associated with increased interdialytic intervals. For instance, one study found a statistically significant increase in risk of sudden death in hemodialysis patients on Mondays and Tuesdays, the period of time during which patients on typical hemodialysis schedules are in their longest interdialytic interval [5]. A separate study reported significantly increased risk of hospitalization for cardiovascular disease in hemodialysis patients at longer times since their last treatment [6]. These and similar results suggest that more frequent dialysis could reasonably be expected to mitigate risk of morbidity and mortality in kidney failure patients, a theory also supported directly by literature [7,8].

Furthermore, such a high degree of toxin clearance is necessary that hemodialysis devices must be quite large to account for the necessary membrane area and pumping apparatus. These devices are largely stationary, and thus immobilize the patient during use, typically for approximately three-to-five hours per day, three days per week in hemodialysis centers. In-home hemodialysis techniques exist and have gained some popularity; however, the patient remains tethered to a large device regularly, and is at increased risk for hospitalization due to infection [9].

To address the limitations of the current hemodialysis regimen, several groups have proposed designs for a “wearable artificial kidney” which acts as a continuous dialyzer without immobilizing the patient [10]. Such devices may represent the future of hemodialysis, but at present still face technical challenges associated with miniaturization without severe losses in transport efficiency. Thus, we have proposed the application of ultrathin nanoporous membranes (nanomembranes) as an ultra-efficient membrane to minimize the area required for dialysis [11].

Nanomembranes were originally developed from pure silicon by Striemer *et al.* with thicknesses on the scale of tens of nanometers [12]. The extraordinary thinness of these membranes enhances transmembrane diffusion by orders of magnitude compared to traditional hemodialysis membranes, which are on the scale of 100 times thicker [13]. Our premise is that this increased transport should reduce the membrane area required to achieve a target solute clearance and thus pave the way for extremely compact, continuous hemodialysis devices.

In this report, we develop analytical and computational models to examine the potential benefits of ultrathin nanomembranes in hemodialysis. Our models describe flow-counterflow dialysis in microfluidic devices featuring nanomembranes. Assuming the steady introduction of newly generated uremic toxins into the blood, we demonstrate adequate clearance of an array of toxins to establish a theoretical proof of concept. Our work suggests that nanomembranes offer a promising avenue for development of novel small-format hemodialysis devices, and that our models will be valuable tools in the design of such devices.

## 2. Experimental Section

### 2.1. Silicon Nanomembranes

The silicon nanomembranes developed by Striemer *et al.* [12] offer an array of characteristics that make them suitable for hemodialysis. They are also highly porous and exceedingly thin, resulting in extremely low resistance to transmembrane diffusion of molecules much smaller than the average pore diameter (on the order of tens of nanometers). Our recent publication describes the creation of Silicon Nitride nanomembranes [14]. The membranes are significantly stronger and more chemically inert compared to the pure silicon nanomembranes developed by Striemer *et al.* [12].

For our models, we consider the membrane to act as a perfect wall for convective flow (prevents convective coupling between the flows on either side [15]) and a uniform region for diffusion in the transmembrane direction only that does not adsorb solutes.

### 2.2. Analytical Model

**Figure 1:**
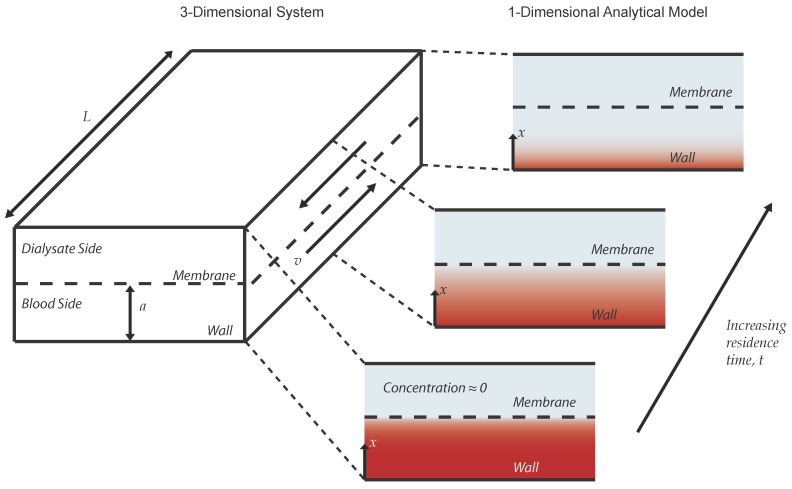
Diagram of system considered by the analytical models. The system is initially at some constant concentration (depicted as red shading) and becomes less concentrated in a gradient over time. Mathematically, the system is one-dimensional; however, it is shown here with a second dimension to illustrate the relationship between the temporal and spatial evolution of the concentration profile.

Our models were designed to predict the fraction of solute that is cleared from a volume of blood as it passes through the dialyzer. The dialyzer system consists of blood flowing through a wide channel, bounded on one side by an impermeable wall and on the other by the nanomembrane (Figure 1). For our first model, we considered a simplified system constrained by several assumptions: (1) the velocity of the blood is constant across the channel (plug flow); (2) diffusive transport of solutes in the direction of flow is negligible compared to convective transport; (3) the concentration of any blood solute of interest in the region beyond the membrane is always zero (a perfect sink). The first two of these assumptions taken together mean that diffusion occurs in only one dimension (towards or away from the membrane) and over an interval of time given by the ratio of the membrane length in the direction of flow to the flow velocity (overall dimensions of time), which is the total residence time for a fluid element in the active region of the channel. The third assumption is founded on the fact that fresh dialysate is constantly washing away the solute beyond the membrane. This will find important application as a boundary condition shortly.

These assumptions allow us to describe the concentration of the solute within the dialyzer as a function of only two independent variables: the distance between the wall and the membrane *x*, and the local residence time of a fluid element in the channel, *t*. A schematic of this system is depicted in Figure 1. Our model is then constructed mathematically from Fick’s second law in one dimension,

(1) $$\frac{\partial c}{\partial t}=D\frac{\partial^{2}c}{\partial x^{2}}$$

where *x* is the location along the axis of diffusion and *c* represents the concentration of the solute of interest at a given *x* and *t*. Since the solute is bounded within the channel on one side by the membrane and on the other by an impermeable wall, this equation can be rewritten non-dimensionally as:

(2) $$\frac{\partial \bar{c}}{\partial \bar{t}}=\frac{\partial^{2}\bar{c}}{\partial {\bar{x}}^{2}}\;\;for\;\;0\leq \bar{x}<1\;\;and\;\;\bar{t}\geq 0$$

where

(3) $$\bar{x}=\frac{x}{a};\;\bar{c}=\frac{c}{c_{0}};\;\bar{t}=\frac{tD_{0}}{a^{2}}$$

where *a* is the distance between the membrane and the wall, $c_{0}$ is the initial concentration of the solute, and $D_{0}$ is the free diffusion coefficient of the solute within the solvent. Assuming that the membrane poses no resistance to diffusion of the solute, we can apply three boundary conditions to Equation (2):

(4) $$\frac{\partial \bar{c}}{\partial \bar{x}}\left(0,\bar{t}\right)=0$$

(5) $$\bar{c}\left(\bar{x},0\right)=1$$

(6) $$\bar{c}\left(1,\bar{t}\right)=0$$

Boundary Condition 4 represents the no-flux condition of the solute through the wall and Condition 5 is the initial concentration of solute. Condition 6 defines the membrane as a sink for the solute. Conceptually, this means that solute that reaches the membrane is swept away by the flow of the dialysate (assumption (3) from earlier,) and that transport across the membrane is unrestricted and instantaneous. Both of these assumptions will be revisited. The solution to this problem is [13]:

(7) $$\bar{c}\left(\bar{x},\bar{t}\right)=\frac{2}{\pi }\sum_{n=1}^{\infty }\frac{{\left(-1\right)}^{n+1}}{n-\frac{1}{2}}cos\left(\lambda_{n}\bar{x}\right)e^{-\lambda_{n}^{2}\bar{t}}$$

where

(8) $$\lambda_{n}=\pi \left(n-\frac{1}{2}\right)$$

Alternatively, if we consider that the membrane *does* resist diffusion across it, we can quantify the contribution of the membrane to the overall diffusive resistance:

(9) $$\beta =\frac{d/D_{m}}{a/D_{0}}$$

where *d* is the thickness of the membrane and $D_{m}$ is the effective diffusion coefficient of the molecule within the membrane space, as calculated by the pore hindrance model in Snyder *et al.* [13]. Due to the extreme thinness of nanomembranes *β* will be very small when considering the transport of small molecules (e.g., urea or creatinine,) but may become significant for larger protein solutes. We can incorporate this into the analytical model by redefining Boundary Condition Equation (6) as:

(10) $$\bar{c}\left(1,\bar{t}\right)+\beta \frac{\partial \bar{c}}{\partial \bar{x}}\left(1,\bar{t}\right)=0$$

This expression simplifies to Equation (6) in the case that β→0, and in such a case the solution in Equation (7) is valid. Otherwise, the concentration profile is given by:

(11) $$\bar{c}\left(\bar{x},\bar{t}\right)=\sum_{n=1}^{\infty }C_{n}cos\left(z_{n}\bar{x}\right)e^{-\lambda_{n}\bar{t}}$$

where $\lambda_{n}=z_{n}^{2}$ and

(12) $$C_{n}=\frac{2sin(z_{n})}{z_{n}+sin\left(z_{n}\right)cos\left(z_{n}\right)}$$

The values of $z_{n}$ are the solutions to the eigenfunction,

(13) $$tan\left(z_{n}\right)=\frac{1}{\beta z_{n}}$$

where $z_{1}$ is the smallest absolute value of *z* that is a solution to Equation (13), $z_{2}$ is the next smallest, and so on.

Whether Equation (7) or Equation (11) is appropriate, the fraction of solute remaining in the blood channel at the outlet for a given time, channel height, and diffusion coefficient can be obtained by averaging the value of $\bar{c}$ over *x*. Fractional clearance can be obtained by subtracting this value from one. In this work, Equation (7) is used unless otherwise stated.

### 2.3. Experimental System and the Finite Element Model

**Figure 2:**
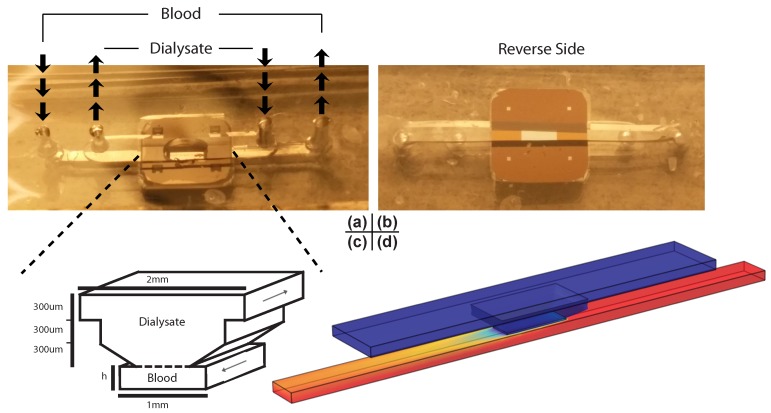
Photos, schematic, and COMSOL finite element model of the experimental system. (**a**) Photo of the experimental system taken from the access side. The dialysate channel passes over the membrane (in the center of the chip) while the blood channel passes underneath it; (**b**) Photo of the same system taken from the other side. The four squares in the corners of the chip in the photographs are additional regions of membrane, but are not in use during this particular experiment; (**c**) Schematic of active section of experimental system. The membrane spans 70% of the blood channel’s width, and the dialysate and blood are in counterflow. The dashed line represents the membrane; (**d**) Snapshot of the finite element model. It is colored with an example heat map representing concentration of a solute as it moves through the system, where the highest concentration is bright red while the lowest is dark blue.

In order to test our models, we built a simple microfluidic device consisting of two channels cut into silicone, separated by a chip supporting the nanomembrane. Photos of the system are shown in Figure 2. On one side of the 300-micrometer-thick chip, the silicon substrate is etched at a 55 degree angle, creating a trapezoidal prism in which the bottom face is the free-standing nanomembrane; on the other side, the membrane is flush with the surface of the chip (Figure 2b, “Reverse Side”). On the dialysate side 1X phosphate buffered saline (pH 7.4) was pumped into a 300-micrometer-tall, 2-millimeter-wide channel connected to the trapezoidal channel at 9 cubic millimeters per second. On the blood side, the same buffer dosed with a known concentration of the experimental analyte was pumped in the opposite direction. The blood channel was 1 millimeter in width and with height adjusted with the volumetric flow rate to keep the average velocity of the fluid within the channel at 0.2 millimeters per second (0.1, 0.3, and 1.0 millimeters channel height for 0.02, 0.06, and 0.20 cubic millimeters per second flow rate, respectively). The membrane dimensions are 700 micrometers in width and 2 millimeters in length, such that the residence time in the blood channel was 10 s. Samples from the blood side were collected and assayed for the concentration of the analyte post-dialysis, using an appropriate assay (BioVision Urea Colorimetric Assay Kit (catalog #K375-100) for urea; absorbance at 410 nanometers for cytochrome c; ThermoFisher Scientific Quant-iT Protein Assay (catalog #Q33210) for bovine serum albumin). For this design, the predictions made by the analytical model are reduced to 70% to account for the fact that only 70% of the blood channel under the membrane in the experimental system is actually exposed to it.

In addition, we constructed finite element models of this system in three dimensions using the COMSOL Multiphysics 4.2a software package. The models consist of three domains: the blood channel, the dialysate channel, and the membrane separating them. Within the channels, fluid flow driven by a known inlet velocity is solved and then passed to the diffusion model as a convective term, where it is combined with a diffusion term to acquire a complete description of solute transport. The membrane is modeled as a purely diffusive space, in which diffusion is allowed to occur in the transmembrane direction only. The value of the finite element models are twofold: first, they can be used to assess the validity of the simpler analytical model, and second, they can be made to incorporate non-ideal three-dimensional geometries as shown in Figure 2 to make accurate predictions of real experimental outcomes. In order to meet the first goal, a very simple model was constructed that is a three-dimensional extrapolation of the one-dimensional system the analytical model describes. If the analytical model is a valid representation of the behavior of three-dimensional systems, a simple rectangular prism should recapitulate the behavior of the analytical model for a given channel height, time, and diffusion coefficient. Note that the COMSOL finite element model is a full simulation of laminar Navier-Stokes fluid dynamics and mass transport that relaxes all of the simplifying assumptions (1–3) of the analytical model for a more realistic description of the system behavior.

### 2.4. Prediction of Dialyzer Adequacy

Once the fractional clearance value for a given dialyzer design and toxin is obtained from the models, a simple analysis allows us to predict the effect the dialyzer would have on the concentration of a particular toxin during continuous treatment. This steady-state concentration of a given toxin can be acquired from a statement of balance between toxin generation and toxin clearance:

(14) $$\dot{G}=\dot{Q}\times C\times f$$

where $\dot{G}$ is the rate of solute generation in amount per time, $\dot{Q}$ is the volumetric flow rate of blood through the dialyzer in volume per time, *C* is the steady-state concentration of solute in amount per volume, and *f* is the fractional clearance of solute from the dialyzer.

### 2.5. Design Selection

Guided by calculations of steady-state concentrations of various uremic toxins for a given dialyzer design, the analytical model can be used to select for reasonable combinations of parameters in order to assess the effectiveness of nanomembranes in miniaturized dialysis. Designs are selected by: (1) iteration through a wide number of combinations of dialyzer geometry, membrane pore size, and blood flow rate; (2) narrowing these down only to systems that result in desired clearance rate of urea and albumin; and finally (3) selecting the system that clears middle molecule toxins most effectively. This algorithm results in a well-rounded dialyzer, but by no means guarantees the best possible result by more heuristic measurements of dialysis quality—it is only a proof of principle. For this reason, an active membrane area of 9 square centimeters was selected due to its practicality. Membranes on this scale represent an approximate upper limit on the size of single contiguous membrane area currently achievable in the laboratory [16].

While the analytical model allows for quick comparison among and selection of designs, it makes no consideration of the practical constraints of real systems. Once a theoretical design has been selected by the analytical model, finite element models incorporating those parameters into more practical designs (*i.e.*, designs that could be fabricated in a laboratory setting) can be constructed and compared. This process serves as our most efficient path for the rapid design and prototyping of dialysis devices.

## 3. Results and Discussion

### 3.1. Validation of Models

**Figure 3:**
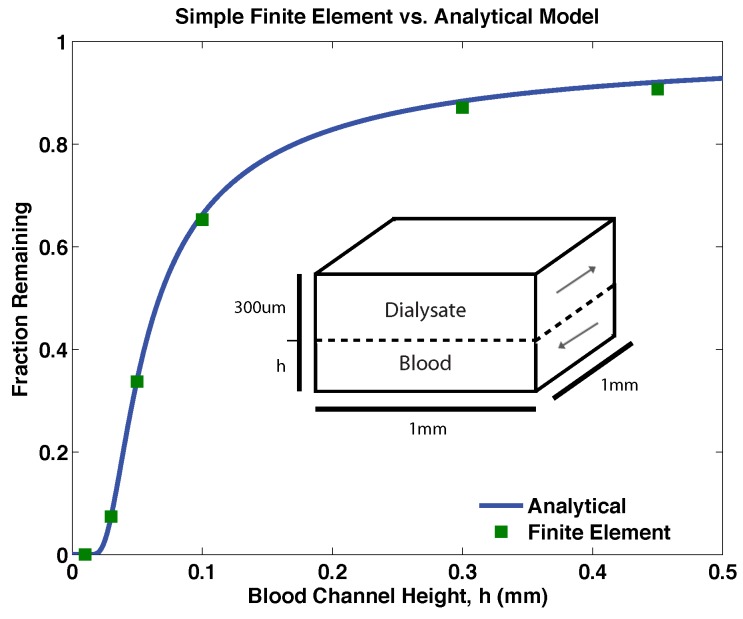
A comparison between the analytical model and a simple finite element model incorporating ideal geometry (schematic inset). Though the analytical model is one-dimensional and the finite element model is three-dimensional, they are in near-exact agreement regardless of blood channel height. These data for a solute of hydrodynamic radius 1.5 nanometers and a blood flow rate of 10 millimeters per minute.

**Figure 4:**
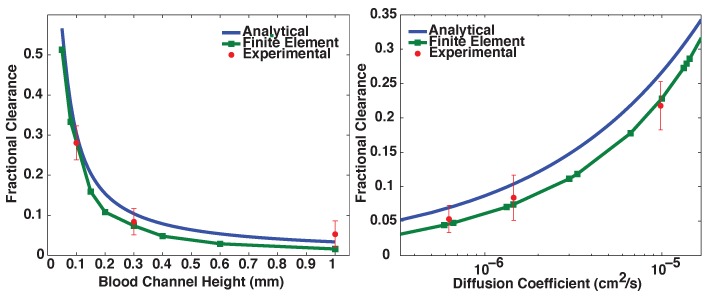
Comparison between the analytical model, the complex geometry finite element model, and experimental results. Experiments were performed with *n* = 3 or 4, and error bars represent standard error of the mean. Experiments varying blood channel height used solute with diffusion coefficient $1.6\times 10^{-6}$ and experiments varying diffusion coefficient used blood channel height of 0.3 mm. Residence time of blood channel fluid near the membrane was 10 s in all cases. The finite element model was highly predictive of experimental results, while the analytical model captured trends but systematically over-predicted clearance.

As expected, the analytical model and the finite element model agree with a high degree of accuracy for simple rectangular geometries (Figure 3). This comparison validates both models and suggests that at least under these flow conditions any difference between the analytical model and finite element model is a product of the complexities of the dialyzer geometry alone. Experimental results were adequately predicted by the finite element models for a range of blood channel heights and solute diffusion coefficients, as shown in Figure 4. The analytical model systematically over-predicts clearance in this design by a small degree because of its assumption of ideal geometry.

### 3.2. Proof-of-Principle Design

**Figure 5:**
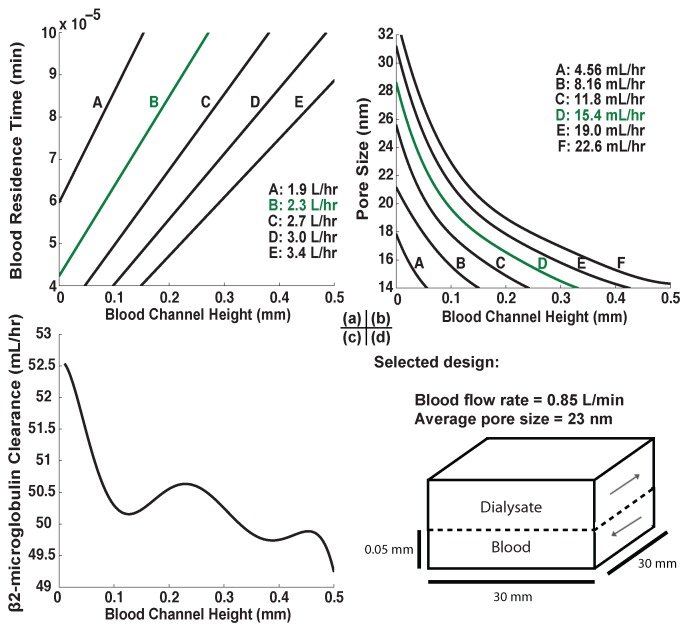
Dialyzer optimization process. Volume per time values in plots (**a**–**c**) are products of the volumetric flow rate of blood and the fractional clearance of urea, albumin, and $\beta_{2}$-microglobulin, respectively. Curves shown are polynomial fits used to express all values as a function of channel height only. (**a**) Isolines of urea clearance with changing blood channel height and blood residence time; line B (green) is the isoline where urea clearance is at the desired value; (**b**) Isocurves of albumin clearance with changing channel height and pore size, with residence time calculated as a function of channel height in order to maintain constant urea clearance. Curve D (green) is the desired curve in this plot; (**c**) $\beta_{2}$-microglobulin clearance as a function of channel height with constant urea and albumin clearances. The shape of this relationship is complex owing to the simultaneous change of three separate variables represented by changes in blood channel height (channel height itself, in addition to blood residence time and pore size); (**d**) Final proof-of-principle design selection. While a channel height of 0.05 millimeters does not correspond to the global maximum of $\beta_{2}$-microglobulin clearance, it is a good compromise between clearance and practicality.

The analytical model was used as a tool for selecting a proof-of-principle design from amongst a wide range of possible combinations of blood flow rates, blood channel heights, and pore sizes by restricting the design in successive steps. First, the active membrane area is set at a reasonable value of 9 square centimeters. Urea clearance is fixed at its target value (Table 1), which constrains the design to a specific relationship between blood channel height and blood flow rate as shown in Figure 5a. Urea is so small that the membrane is unable to hinder its diffusion significantly (*i.e.*, *β* is always very small), so pore size is ignored in this step. Next, given this relationship we fix the clearance of the large molecule albumin to its desired value to find a relationship between pore size and channel height (Figure 5b). Finally, given pore size and blood flow rate as functions exclusively of channel height, the clearance of middle molecule $\beta_{2}$-microglobulin is evaluated as a function of channel height only and maximized (Figure 5c).

**Table 1:** Generation rates $\dot{G}$, physiological concentrations, target clearance rates, and predicted steady-state concentrations during continuous nanomembrane dialysis for selected uremic toxins as well as albumin. "Physiological concentrations" refer to reference concentrations expected of patients in good health. Values may vary from patient-to-patient and serve here only as approximations of averages for a mean body weight of 70 kg. An example target clearance calculation is supplied in Equation (15). Steady-state concentrations are predicted using Equation (7) for β<0.2 (see Equation (9)) and (11) otherwise.

Plasma Solute	$\dot{G}$ (mmol/h)	Physiological Concentration (mM)	Target Clearance (L/h)	Steady-State Concentration (mM)
		4.6 [18]	2.3	4.6
Creatinine	0.58 [19]	0.11 [18]	5.3	0.29
$\beta_{2}$-microglobulin	$7.7\times 10^{-4}$ [20]	$1.5\times 10^{-4}$ [21]	5.1	$1.5\times 10^{-2}$
Albumin	$1.0\times 10^{-2}$ [22]	0.65 [18]	$1.5\times 10^{-2}$	0.65

Desired clearance can be obtained from the required product of fractional clearance and volumetric flow rate for a given solute. For instance, for urea:

(15) $$\dot{Q}\times f_{urea}=\frac{{\dot{G}}_{urea}}{C_{urea}}=2.3\frac{L}{h}$$

where $C_{urea}$ is the reference physiological concentration serum of urea in a healthy adult. When this equation is satisfied, the dialyzer maintains plasma urea at $C_{urea}$, so for adequate urea clearance, the left hand side of the equation must equal at least 2.3 L·h${}^{-1}$. The maximum possible value of fractional clearance *f* is of course 1, so the minimum possible $\dot{Q}$ is 2.3 L·h${}^{-1}$. However, for any feasibly miniaturized design, the corresponding $f_{urea}$ for this minimum $\dot{Q}$ is significantly less than 1; thus, $\dot{Q}$ must be increased to compensate, in turn reducing $f_{urea}$ further (but by a smaller degree than the increase in $\dot{Q}$), and so on. The product of these values begins to approach the desired 2.3 L·h${}^{-1}$ only at rather high flow rates.

The design selected for proof of principle by this process (Figure 5d) has an active membrane area of just 9 square centimeters, a blood channel height of 50 micrometers, and passes blood at a rate of 0.85 liters per minute. Its nanomembrane has an average pore diameter of roughly 23 nanometers, and these factors combine to produce the steady-state concentrations shown in Table 1. It is noteworthy that while the urea and albumin clearances are ideal by design, the maximum possible $\beta_{2}$-microglobulin clearance rate is fully two orders of magnitude lower than the target value. This will be addressed further.

### 3.3. Finite Element Models of Practical Designs

**Figure 6:**
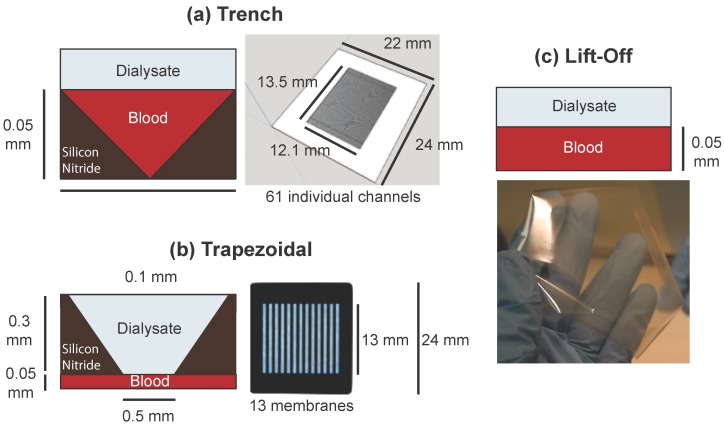
Depictions of the practical designs referenced in Table 2. (**a**) The trench chip design, consisting of a series of triangular channels etched under a continuous membrane. Sixty-one individual channels are fed by a single inlet and empty into a single outlet, and 11 such chips would be required for a total active membrane area of 9 square centimeters; (**b**) The trapezoidal chip design, incorporating 13 regularly spaced membranes instead of a single continuous membrane. Fourteen chips are needed to reach 9 square centimeters of membrane area. While in the original design used by Johnson *et al.* the trapezoidal windows were the blood channels with dialysate on the other side, this was reversed here due to the need for such thin blood channels; (**c**) The lift-off design. This free-standing membrane can be fabricated up to approximately 9 square centimeters in a single sheet and would need to be held up by periodically placed supports; however, these would not affect the clearance characteristics.

We are now ready to make predictions of solute clearance in realistic geometries. We consider several device designs that have been developed or are under development in the laboratory: a chip with many shallow triangular trenches etched into it beneath the membrane (the “trench” design, Figure 6a); a chip with 13 trapezoidal membrane windows resting over a thin channel as demonstrated by Johnson *et al.* [11] (the “trapezoidal” design, Figure 6b); and a uniform sheet of membrane as demonstrated by Miller *et al.* [16], suspended over a set of thin channels (the “lift-off” design, Figure 6c). The results of these simulations are shown in Table 2.

**Table 2:** Comparison between target physiological concentrations of various toxins and steady-state concentration results from three-dimensional finite element models of practical geometries depicted in Figure 6. Values are steady state concentrations in mM. While the trapezoidal and lift-off designs have too much clearance of albumin and not enough of smaller solutes, the trench design achieves a more desirable distribution of steady state concentrations.

Plasma Solute	Physiological	Trapezoidal	Trench	Lift-off
Urea	4.6	24	2.1	5.7
Creatinine	0.11	1.8	0.14	3.9
$\beta_{2}$-microglobulin	$1.5\times 10^{-4}$	$1.3\times 10^{-2}$	$6.0\times 10^{-4}$	$2.3\times 10^{-3}$
Albumin	0.65	7.0	$2.0\times 10^{-2}$	0.10

Note: All values in mM.

While the lift-off design straddles the line between clearance that is too low (for smaller molecules) and clearance that is too high (for larger molecules) and the trapezoidal design loses a large degree of efficiency due to its significant inactive area, the trench design has a more desirable relationship between solute size and clearance rate. It also provides a much-needed boost to middle-molecule $\beta_{2}$-microglobulin clearance over the analytical model, apparently sharpening the size-based cutoff characteristics of the design.

We concluded, then, that the triangular channel design of the trench chips enhances the selectivity of the system for clearing small molecules with the same volumetric flow rate. This may be owing to the enhanced diffusivity of small molecules laterally within the blood channel: all solutes will be more rapidly depleted near the corners of the triangular channel due to reduced distance between wall and membrane, but the increased diffusivity of smaller molecules enables them to more quickly spread out into the corners to replace this depletion, and thus take advantage of the corners more readily. Investigation into the advantages this design offers may prove invaluable as our research progresses further.

### 3.4. Discussion

Traditionally, dialysis adequacy is measured by the dialyzer’s clearance of a marker solute, most often urea or creatinine. While extensive research has been conducted on these metrics (often by empirical correlation with patient outcome), more careful analysis of the co-morbidities of kidney disease reveals that small molecule markers are not major contributors to uremic toxicity. Instead, toxicity is primarily exercised by buildup of middle molecules and protein-bound toxins, such as $\beta_{2}$-microglobulin, *p*-cresyl sulfate, and indoxyl sulfate, the latter two of which build up in kidney failure patients due to high affinity for serum albumin, which is not substantially cleared by most hemodialysis techniques [4,23]. It is for this reason that our design was selected with middle and large molecules in mind.

The ability of a miniaturized nanomembrane dialyzer to achieve steady-state concentrations of uremic toxins close to healthy physiological values (Tables Table 1 and Table 2) suggests that this device design approximates the size-based clearance characteristics of a healthy kidney fairly well. This represents a dramatic improvement over traditional dialysis, which fails to adequately clear many uremic toxins larger than urea. For instance, one study found serum $\beta_{2}$-microglobulin concentrations roughly 45 times higher in hemodialysis patients, compared to control participants [24]. Similarly, albumin-binding toxin *p*-cresyl sulfate has been found to be elevated by as much as three times over normal in late-stage chronic kidney disease patients in spite of frequent hemodialysis, likely due to poor clearance of serum albumin during traditional treatments [25].

It’s fortunate that finite element modeling of the trench chip design resulted in sufficient clearance of $\beta_{2}$-microglobulin given that the analytical model predicted very poor clearance of that toxin for systems with ideal geometry. However, it is probable that a more desirable set of clearance characteristics is possible with the analytical model if the restriction of a specific urea clearance rate is relaxed. Given that the elevation of serum $\beta_{2}$-microglobulin and other middle molecules is so much more of a contributing factor to overall uremic toxicity than urea itself, future designs may benefit from addressing the clearance rate of middle molecules preferentially, and allowing small molecules to remain somewhat elevated.

It is notable that the lift-off design did not result in ideal clearance characteristics despite its ideal geometry. This deviation has two causes: first, the assumption of plug flow made by the analytical model becomes an increasingly worse approximation at higher flow rates. Second is the exacerbation of small errors in fractional clearance caused by high flow rates. That is to say that the magnitude of the change in the steady-state concentration *C* derived from Equation (14) due to a small change in *f* is scaled by the value of $\dot{Q}$. Thus even a deviation from ideal fractional clearance can have significant effects on the resulting serum concentration of toxins. This line of reasoning favors lower flow rates in dialyzers, and must remain in consideration as design efforts progress.

High blood flow rate is generally considered undesirable in hemodialysis devices which access blood via arteriovenous fistula, owing to increased demand on patients’ hearts. However, studies attempting to qualify the amount of arteriovenous access flow which is dangerous have reported that there is little cardiac concern for patients with otherwise healthy hearts for access flow rates less than approximately 30% of their total cardiac output (reference range 4 to 8 L·min${}^{-1}$) [26]. Thus, this device would likely pose little risk to patients healthy enough for access via arteriovenous fistula due to its 0.85 L·min${}^{-1}$ blood flow rate.

Interestingly, the small active area required for a miniaturized nanomembrane dialyzer could feasibly obviate the need for pumping of blood, instead relying on the patient’s natural arteriovenous pressure drop to supply the necessary flow. For instance, if the 9 square centimeter active membrane area of the device described above is 30 centimeters perpendicular to the direction of flow and 0.3 cm parallel to it, rough estimation of the device’s hydraulic resistance reveals that 14,000 Pascals (an estimation of mean arteriovenous pressure drop) applied across the device is sufficient to achieve about 1 L·min${}^{-1}$ of blood flow [27]. While this is probably an undesirable design for actual implementation, it illustrates the benefit of inventive geometrical designs. Devices on this scale may be able to take advantage of low hydraulic resistance to further reduce overall dialyzer size by removal of the blood pump.

Another advantage of miniaturized dialysis is reduced extracorporeal blood volume. The minimum necessary blood volume for the 9 square centimeter device is just 45 microliters, compared to between 47 and 121 milliliters for traditional hollow fiber dialysis machines (not to mention a significant volume of tubing between the fibers and the patient) [28]. This reduction in volume could be reasonably expected to alleviate some of the risk of hypotension and hypothermia during treatment; however, this will also depend on the degree of ultrafiltration, which will in turn depend on the characteristics of the dialysate channel. Reduced volume also reduces the risk associated with accidental disconnection from the device during use, as less blood is lost in the process—a factor that may be as important in prompting patients to adopt the device as in keeping them safe.

## 4. Conclusions

Ultra-permeable silicon membranes have been proposed as an enabling technology for miniaturized continuous hemodialysis devices, which could offer improved treatment for people with end stage or chronic renal disease. Our models strongly suggest that miniaturized nanomembrane dialysis is not only feasible, but highly desirable due to our predictions of highly effective control of uremic toxin levels via this technology. Future work should involve experimental prototypes of designs predicted by our models to be effective dialyzers, with benchtop experiments to confirm predictions and animal trials to assess the devices’ efficacies as treatments for the symptoms of renal failure.
